# Association of service use with subjective oral health indicators in a freedom of choice pilot

**DOI:** 10.1002/cre2.680

**Published:** 2022-10-20

**Authors:** Eero Raittio, Eeva Torppa‐Saarinen, Taru Sokka, Satu Lahti, Auli Suominen, Anna Liisa Suominen, Anna Maria Heikkinen

**Affiliations:** ^1^ Oral Health Care Services Tampere Finland; ^2^ Institute of Dentistry University of Eastern Finland Kuopio Finland; ^3^ Department of Dentistry and Oral Health Aarhus University Aarhus Denmark; ^4^ The Council of Tampere Region Tampere Finland; ^5^ Department of Community Dentistry University of Turku Turku Finland; ^6^ Oral Health Teaching Clinic, Kuopio University Hospital Kuopio Finland; ^7^ Faculty of Medicine and Health Technology Tampere University Tampere Finland

**Keywords:** delivery of health care, dental anxiety, dental health services, oral health‐related quality of life, oral health

## Abstract

**Objectives:**

A freedom of choice pilot provided access to private oral health care services without queuing and with fixed public service‐fees for participants in Tampere region, Finland in 2018–2019. The aim of this study was to investigate how use of oral health care services differed by demographics, socioeconomic status, dental fear, and self‐reported oral health in this pilot.

**Material and methods:**

SMS‐messages including a link to online questionnaire were sent to participants who had booked an appointment, and to those who had not booked an appointment despite registering to pilot. We categorized participants to (1) those who had booked their first appointment before receiving SMS (*visitors*), (2) those who booked an appointment after receiving the SMS‐message (*late‐visitors*), and (3) those who had not booked an appointment during pilot (*nonvisitors*). We used regression analysis to estimate the association of age, gender, dental fear, economic situation, Oral Health Impact Profile‐14‐severity (oral health‐related quality of life [OHRQoL]), self‐reported oral health and need for oral health care (exposures) with oral health care service use during the pilot (outcome).

**Results:**

Out of 2300 participants, 636 (28%) responded. *Late‐visitors* were more likely older and reported more likely need for oral health care, poorer oral health and OHRQoL than *visitors* or *nonvisitors*. *Nonvisitors* were younger and had better OHRQoL than the others. The differences in the service use by gender, economic situation, and dental fear were small.

**Conclusions:**

Service use during the pilot depended on the subjective oral health. Our findings highlight the potential of reminders in increasing care use among those with perceived need for services.

## 1 INTRODUCTION

In Finland the whole population has been entitled to use subsidized oral health care services since December 2002. Since then, the entire Finnish population has been entitled to use public oral health care services (POHCS). Alternatively, they are entitled to receive reimbursements from the National Health Insurance for their private oral health care costs. From a user perspective, POHCS, which are maintained by municipalities (309 in 2021, in Finland), provide much cheaper services with nationally fixed out‐of‐pocket fees for adults. In addition to the basic fee, there are separate procedure fees for treatments and examinations depending on complexity class. In the private sector, client fees are not fixed and the reimbursement level from the National Health Insurance has long been decreasing. Additionally, private services are found mainly in the large municipalities, whereas POHCS are available in every municipality. As adults have freedom to choose the service sector they use, unsurprisingly the demand for and use of subsidized oral health care services has been uneven between the sectors since the reform. In many municipalities, POHCS has been congested which has led to long treatment queues and prolonged treatment periods. Meanwhile, in the private sector there is considerable overcapacity in certain areas. However, the majority of private dentists feel that the demand for their care is reasonable, thus guaranteeing significantly faster access to services in addition to better availability of specialist care (Raittio, 2016).

To meet the demand for services and to explore different oral health care provision strategies, the government introduced a freedom of choice oral health care pilot (FCOHC‐pilot) during 2018–2019. It included access to private adult oral health services (excluding acute, implant, and fixed prosthodontic care) with the fixed fees of POHCS. The project was partly financed by the government, partly by the municipalities and out‐of‐pocket payments. The private caregivers in Tampere region showed interest in the project, since 13 service providers joined the pilot. Regular measurements of subjective oral health and patient experience were also part of the pilot.

The connection between the use of oral health care services and oral health is complex. On the one hand, the oral health care service use seems to affect clinical and subjective oral health status, and on the other hand, clinical and subjective oral health status seem to affect the service use (Torppa‐Saarinen et al., 2018, 2019, 2021). It is also well known that out‐of‐pocket payments, dental fear and queues affect the oral health care service use (Cooray et al., 2020; Manning et al., 1985; Mueller & Monheit, 1988; Raittio et al., 2014; Suominen et al., 2017). For instance, considerable proportion of Finnish adults report that out‐of‐pocket payments and queues hinder their oral health care service use (Suominen et al., 2017).

The conducted FCOHC‐pilot improved access to oral health care services without queuing for the pilot participants with the fixed fees of POHCS. Thus, with the available information on the pilot participants' subjective oral health, the pilot served as a fruitful base for investigating how subjective oral health measures and other determinants are connected with the oral health care service use. Particularly, we investigated how those who did or did not book appointments during the pilot differed by their subjective oral health, oral health‐related quality of life (OHRQoL), self‐assessed treatment need, dental fear, and perceived financial situation.

## 2 MATERIALS AND METHODS

In May 2018 4000 adult individuals residing in Tampere region, Finland were recruited to the project by public announcements, and in August 2018, 1000, more were recruited. In addition, 419 extra registrations were allowed to ensure the use of allocated resources. The registration took place online with a program designed for the project, requiring a strong authentication thus verifying the place of residence and eligibility to take part. The participants gave their phone number, chose the private oral health care provider they wanted to use and answered background questions. They were given instructions on how to book their first appointment. The patient records were electronic and the software was provided by the project (City of Tampere, 2019).

The research approval taking into account ethical aspects was granted by the city of Tampere (City of Tampere, 2019). The participants received written information on the research protocol and consented that background information, clinical findings, attendance, and their user feedback on the services could be confidentially used for study.

In the FCOHC, previsit SMS‐messages, which included a link to online questionnaire were sent to pilot participants who booked their first visit before February 13, 2019. In addition, it was sent to those who had not booked an appointment until date February 13, 2019 (reminder SMS‐message).

With these questionnaires we gathered information about subjective oral health, dental fear, and economic situation. Subjective oral health measures included self‐assessed treatment need (yes/no), perceived oral health (good/rather good/moderate/rather poor/poor) and the 14‐question version of Oral Health Impact Profile (OHIP‐14) for the OHRQoL. Economic situation was queried with a question: “How would you describe the current balance between income and expenditure in your household?” with reply alternatives: “We have more than enough money to cover our needs,” “There is enough money to cover our needs,” “We have, to some extent, to compromise when deciding what we do with our money,” “We have to compromise considerably in our consumption but we can manage on our income,” “We have to make major compromises in our consumption and, despite that, we do not manage on our own income” and “I cannot say/it is hard to estimate.” Dental fear was elicited with question: “Do you think that visiting a dentist is…” using reply alternatives “not frightening at all,” “somewhat frightening” and “very frightening.”

Information about age, gender, and appointments during pilot were collected from the oral health care data system.

To measure service use during the pilot, we categorized participants to (1) those who had booked their first appointment before receiving SMS (*visitors*), (2) those who booked an appointment after receiving the SMS‐message (*late‐visitors*), and (3) those who had not booked an appointment during pilot (*nonvisitors*).

### 2.1 Statistical analyses

We used regression analysis to estimate the association of age, gender, dental fear, economic situation, and perceived oral health variables (exposures) on oral health care service use during the pilot (outcome). We first drew our assumptions about the causal relationships of variables in a directed acyclic graph (DAG) using DAGitty (Supporting Information: Figure 1) (Tennant et al., 2021). Because perceived oral health and OHIP are very closely related (Kaprio et al., 2012), perceived oral health and OHIP shared same node in our DAG. Based on our DAG, we identified minimum adjustment sets to estimate total effect (association) of each exposure (node) on the outcome with DAGitty (Table 1). As resulting with the use of this procedure, for each exposure‐outcome pair, we identified all confounders which should be adjusted for and all mediators and colliders which should not be adjusted for (Tennant et al., 2021).

**Table 1:** Minimum adjustment sets according to exposure variables based on a directed acyclic graph (DAG)

Total effect (association) of *X* on oral health seeking	Adjustment based on the DAG
Age	No
Gender	No
Economic situation	Age, gender
Dental fear	Age, gender
Self‐perceived treatment need	Age, gender, economic situation, dental fear
OHIP‐14 severity/OHIP dimensions/Perceived oral health	Age, gender, economic situation, dental fear, self‐perceived treatment need

Abbreviation: OHIP, Oral Health Impact Profile.

Statistical analyses were done in R environment (version 4.0.3) with packages nnet (Venables & Ripley, 2002), DAMISC (Armstrong, 2022), ggplot2 (Wickham, 2016) and tableone (Yoshida & Bartel, 2021). We performed multinomial regression analysis for each exposure‐outcome pair with the adjustment variables. In addition to models with service use as outcome for age, gender, dental fear, economic situation, and perceived oral health care treatment need, we ran separate models for OHIP‐14 severity, seven dimensions of OHIP and perceived oral health with same adjustment variables, and therefore there were 14 regression models in total. Then, we generated average effects for each exposure‐outcome pair. We visualized the average effects of each exposure on outcome. In addition, we produced a table of basic descriptive statistics of our variables.

## 3 RESULTS

**Figure 1:**
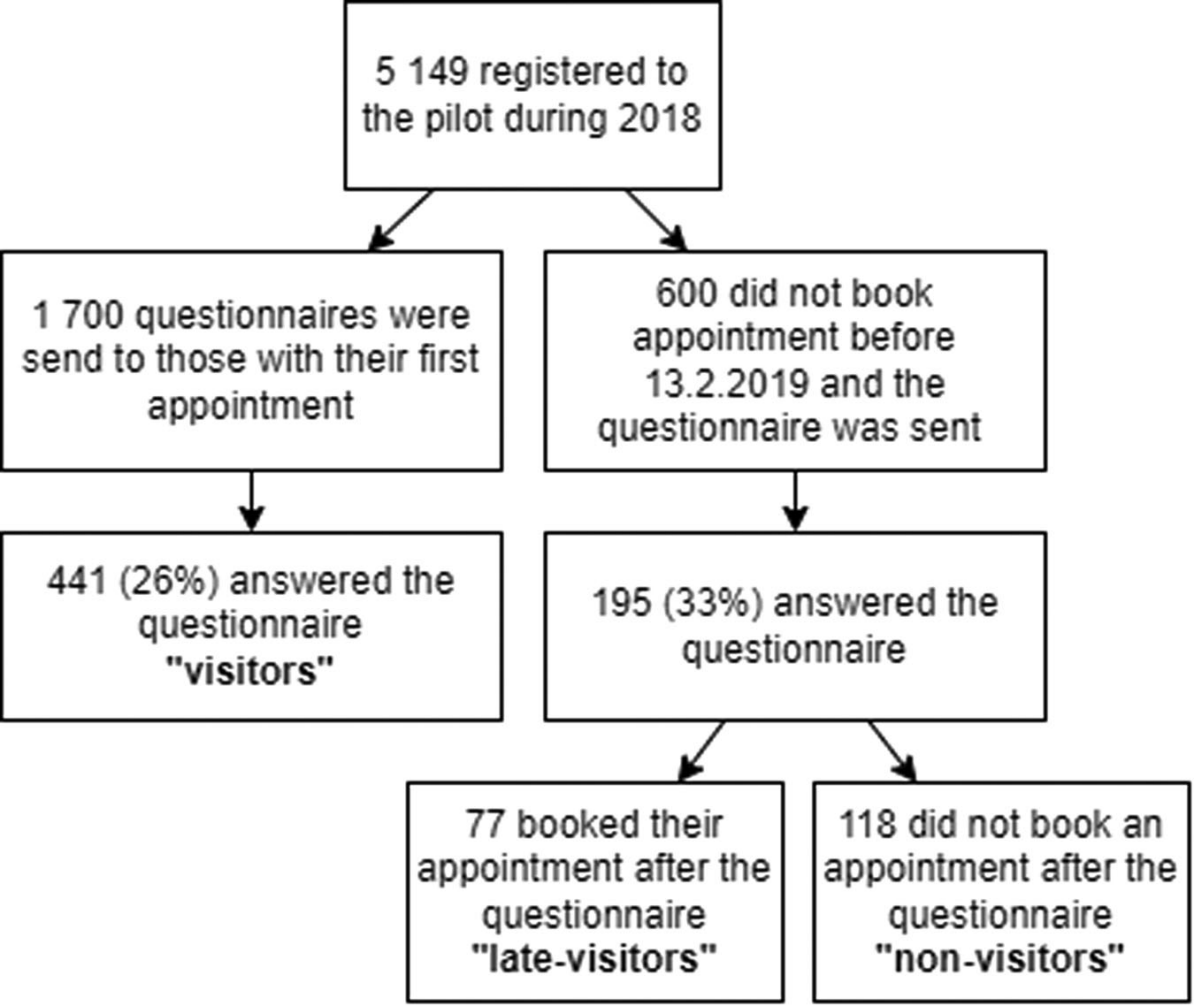
Flowchart of participant recruitment and categorization based on oral health care service use

During 2018, 5419 adults were registered to the pilot (Figure 1). Of those, 1700 who had booked their appointment received a questionnaire, and one‐fourth (26%) completed it (*visitors*). Of 5419, 600 did not book their first appointment, but received the same questionnaire. Of the 195 who answered, 39% booked an appointment (*late‐visitors*) while 61% did not visit at all (*nonvisitors*).

The *late‐visitors* were on average older (57.4 years) and the *nonvisitors* younger (51.1 years) than the *visitors* (54.0 years) (Table 2). The groups were similar according to gender, perceived economic situation, and dental fear. Higher proportion of the *late‐visitors* reported the need for oral health care or had poor or rather poor perceived oral health than of the *visitors* or *nonvisitors*. The *late‐visitors* also reported poorer OHRQoL than the other groups, and rather consistently also in all dimensions of OHRQoL.

**Table 2:** Descriptive statistics of data in total and stratified by oral health seeking

	Total	Visitors	Late visitors	Nonvisitors	*p* Value
*n* (%)	636 (100)	441 (69.3)	77 (12.1)	118 (18.6)	
Age (mean [SD])	53.9 (13.79)	54.0 (13.40)	57.4 (14.03)	51.1 (14.60)	0.007
Gender = women (%)	366 (57.5)	255 (57.8)	41 (53.2)	70 (59.3)	0.688
Economic situation (%)					0.715
Cannot manage with own income	27 (4.2)	18 (4.1)	6 (7.8)	3 (2.5)	
Considerable compromises in consumption	80 (12.6)	61 (13.8)	6 (7.8)	13 (11.0)	
Some compromises in consumption	168 (26.4)	112 (25.4)	21 (27.3)	35 (29.7)	
Enough money to cover needs	258 (40.6)	176 (39.9)	34 (44.2)	48 (40.7)	
More than enough money	70 (11.0)	51 (11.6)	6 (7.8)	13 (11.0)	
Missing data	33 (5.2)	23 (5.2)	4 (5.2)	6 (5.1)	
Dental fear (“Visiting a dentist is…”) (%)					0.224
Not frightening at all	329 (51.7)	234 (53.1)	39 (50.6)	56 (47.5)	
Somewhat frightening	237 (37.3)	155 (35.1)	31 (40.3)	51 (43.2)	
Very frightening	57 (9.0)	39 (8.8)	7 (9.1)	11 (9.3)	
Missing data	13 (2.0)	13 (2.9)	0 (0.0)	0 (0.0)	
Perceived treatment need (%)					0.001
Yes	416 (65.4)	277 (62.8)	65 (84.4)	74 (62.7)	
No	207 (32.5)	151 (34.2)	12 (15.6)	44 (37.3)	
Missing data	13 (2.0)	13 (2.9)	0 (0.0)	0 (0.0)	
Perceived oral health (%)					0.017
Poor	13 (2.0)	7 (1.6)	3 (3.9)	3 (2.5)	
Rather poor	64 (10.1)	44 (10.0)	13 (16.9)	7 (5.9)	
Moderate	239 (37.6)	157 (35.6)	31 (40.3)	51 (43.2)	
Rather good	204 (32.1)	140 (31.7)	26 (33.8)	38 (32.2)	
Good	103 (16.2)	80 (18.1)	4 (5.2)	19 (16.1)	
Missing data	13 (2.0)	13 (2.9)	0 (0.0)	0 (0.0)	
OHIP‐14 (mean [SD])	8.0 (8.60)	8.3 (8.29)	9.9 (10.85)	6.0 (7.69)	0.005
Functional limitation (mean [SD])	0.4 (1.01)	0.3 (0.91)	0.7 (1.50)	0.4 (0.97)	0.022
Physical pain (mean [SD])	2.3 (1.91)	2.4 (1.91)	2.6 (2.17)	1.8 (1.62)	0.002
Psychological discomfort (mean [SD])	1.9 (2.08)	2.0 (2.03)	2.3 (2.35)	1.5 (1.99)	0.011
Physical disability (mean [SD])	0.7 (1.30)	0.7 (1.26)	0.9 (1.74)	0.4 (1.03)	0.014
Psychological disability (mean [SD])	1.2 (1.68)	1.3 (1.65)	1.6 (1.85)	0.9 (1.61)	0.032
Social disability (mean [SD])	0.8 (1.37)	0.8 (1.36)	0.9 (1.62)	0.6 (1.21)	0.116
Handicap (mean [SD])	0.8 (1.28)	0.9 (1.30)	1.0 (1.51)	0.6 (0.99)	0.040

**Figure 2:**
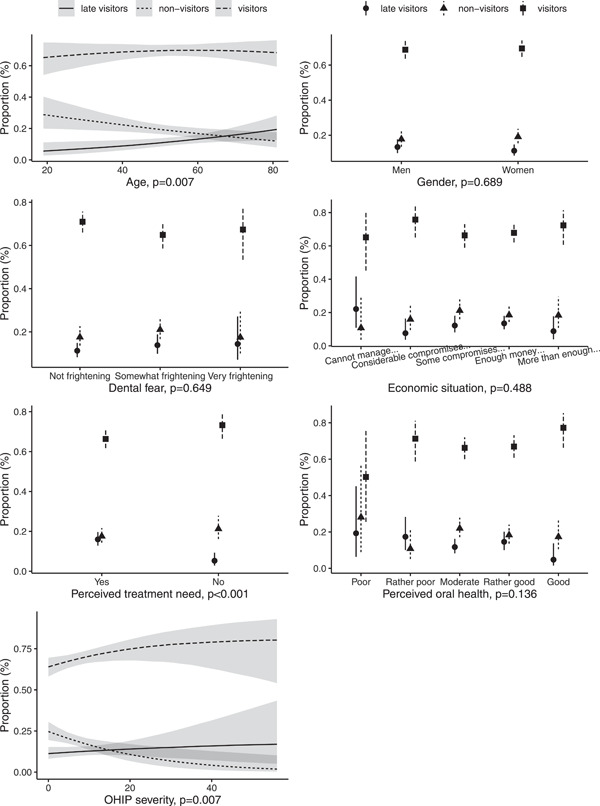
Average effect of each exposure on oral health care service use from multinominal regression analyses

**Figure 3:**
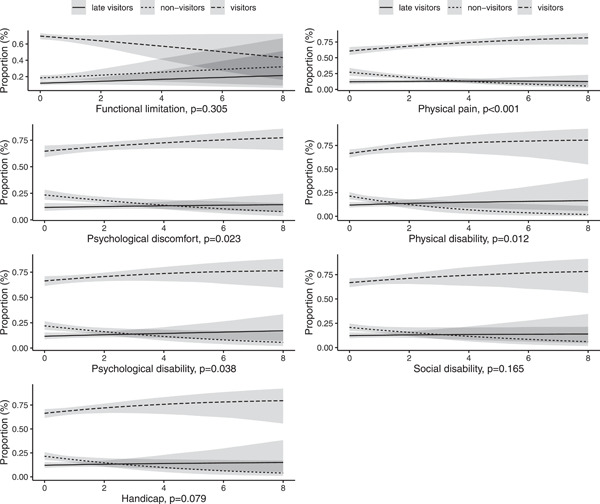
Average effect of each Oral Health Impact dimension on oral health care service use from multinominal regression analyses

When the relationships between the exposures and outcome were adjusted for available confounders (Table 1) with multinomial regression analyses (Figures 2 and 3), the associations were mostly similar to bivariate differences shown in the Table 2. However, the associations of perceived oral health and functional limitation and handicap‐dimensions of OHRQoL with oral health care use were attenuated by the adjustments (Figures 2 and 3).

## 4 DISCUSSION

Our findings indicate that there were some differences in the oral health care service use by age, self‐assessed treatment need, subjective oral health and OHRQoL among the FCOHC‐pilot participants. Those who sought care after the reminder and questionnaire (*late‐visitors*) were more likely older and reported more likely need for oral health care, poorer oral health, and OHRQoL than those who visited or did not visit at all during the pilot. Those who did not visit during the pilot were younger and had better OHRQoL than the others. The differences in the service use by gender, economic situation and dental fear were small.

Taking in to account the literature on the determinants of the oral health care service use in Finland (Nguyen et al., 2005; Suominen et al., 2017; Torppa‐Saarinen et al., 2019) and elsewhere (Hajek et al., 2021), we detected only quite small differences in the service use by self‐reported oral health, dental fear, economic situation, and demographics. Some reasons can be postulated. First, the sample in our study was very selected, because it included only those who were able to register themselves to the pilot and who also responded to the questionnaire. For instance, it may be that those, who have had economic problems and for that reason postponed their visits, did not register to the pilot because the fees were same as in the POHCS already (i.e., one could not receive any cheaper care through the pilot). Second, the highly selected sample may have affected the studied associations because the investigated oral health care service use and its determinants may have affected the registration and responding to the questionnaire and thus the sample selection process, which may in turn have induced biased estimates in our study due to so‐called collider bias (Cole et al., 2010).

Interestingly, despite the highly selected group of individuals, the response rate was quite low. This is consistent with the findings related to quite low general response rates to health care questionnaires directed to patients (Booker et al., 2021). Solutions to increase the response rates for this kind of questionnaires should be looked for in future because they are considered important in evaluation of quality of (oral) health care (Riordain et al., 2021).

Our findings were in line with research (Guy et al., 2012; Kannisto et al., 2014; Schwebel & Larimer, 2018) and experiences from the field showing that reminders and other SMS‐messages increase the likelihood of (a) seeking of care or examination, like cancer screening or vaccination, and (b) attending a scheduled appointment. For instance, Scottish (Perry, 2011), Indian (Prasad & Anand, 2012) and Australian (Storrs et al., 2016) studies have indicated that appointment reminders can considerably decrease missed appointments in oral health care setting. However, some contradicting evidence also about the effects of reminders on the attendance to dental care have been found; the authors have speculated that it has been to high proportion of vulnerable populations and no financial consequence for failing to attend (Bellucci et al., 2017; Stormon et al., 2022). SMS‐messages have also been successfully used to improve compliance to various medical interventions and self‐care, including oral hygiene (Schwebel & Larimer, 2018). Our findings indicate that those who reacted after receiving the reminder (and questionnaire) were older and reported more likely need for oral health care, poorer oral health and OHRQoL. The potential of reminders in increasing the services should not be overlooked as a way of increasing the oral health care service use among those for whom the services are directed to.

Our study is not without limitations. First, the sample size was quite small and based on low response rate, which may undermine validity, reliability, and generalizability of our findings. In addition, the generalizability of our findings is also weak due to the uniqueness of the pilot. Second, we missed some important data. We had only data about care seeking, not about number or content of visits. We also missed important confounders, such as clinically assessed oral health and history of service use. However, they are to some extent represented by the available variables (such as self‐assessed treatment need and subjective oral health measures). On the other hand, we gathered systematically collected data and implemented well‐grounded statistical analyses to answer the question whether those who did or did not book appointments during the pilot differed from each other by their subjective oral health, OHRQoL, self‐assessed treatment need, dental fear, and perceived financial situation.

In conclusion, our study showed that the differences in the use of oral health care services by gender, economic situation, and dental fear were small among the FCOHC‐pilot participants. However, those who sought care after the reminder and questionnaire (*late‐visitors*) were more likely older and reported more likely need for oral health care, poorer oral health, and OHRQoL than those who visited or not visited at all during the pilot. Those who did not visit at all during the pilot were younger and had better OHRQoL than those who visited with or without receiving a reminder. Despite uncertainty caused by the low response rates to the questionnaires, our findings highlight potential of reminders in increasing care seeking among those with perceived need for services.

## AUTHOR CONTRIBUTIONS

**Raittio Eero**: Conceptualization; formal analysis; visualization; writing – original draft preparation. **Torppa‐Saarinen Eeva**: Conceptualization; investigation; funding acquisition; writing – review & editing. **Taru Sokka**: Conceptualization; investigation; writing – review & editing. **Satu Lahti**: Conceptualization; funding acquisition; writing – review & editing. **Auli Suominen**: Data curation, writing – review & editing. **Anna Liisa Suominen**: Conceptualization; writing – review & editing. **Anna Maria Heikkinen**: Conceptualization; investigation; writing – review & editing.

## CONFLICT OF INTEREST

The authors declare no conflict of interest.

## Supporting information

Supporting information.

## Data Availability

The data that support the findings of this study are available from City of Tampere. Restrictions apply to the availability of these data, which were used under license for this study.

## References

[cre2680-bib-0001] Armstrong, D. (2022). *DAMisc: Dave Armstrong's Miscellaneous Functions*. Accessed August 20, 2021. Retrieved from https://CRAN.R-project.org/package=DAMisc

[cre2680-bib-0002] Bellucci, E. , Dharmasena, L. , Nguyen, L. , & Calache, H. (2017). The effectiveness of SMS reminders and the impact of patient characteristics on missed appointments in a public dental outpatient clinic. Australasian Journal of Information Systems, 21. 10.3127/ajis.v21i0.1405

[cre2680-bib-0003] Booker, Q. S. , Austin, J. D. , & Balasubramanian, B. A. (2021). Survey strategies to increase participant response rates in primary care research studies. Family Practice, 38(5), 699–702. 10.1093/fampra/cmab070 34213547

[cre2680-bib-0004] City of Tampere . (2019). Rules of oral health care freedom of choice pilot in the city of Tampere in 2019 (p. 59). Accessed June 10, 2022. City of Tampere.

[cre2680-bib-0005] Cole, S. R. , Platt, R. W. , Schisterman, E. F. , Chu, H. , Westreich, D. , Richardson, D. , & Poole, C. (2010). Illustrating bias due to conditioning on a collider. International Journal of Epidemiology, 39(2), 417–420. 10.1093/ije/dyp334 19926667 PMC2846442

[cre2680-bib-0006] Cooray, U. , Aida, J. , Watt, R. G. , Tsakos, G. , Heilmann, A. , Kato, H. , Kiuchi, S. , Kondo, K. , & Osaka, K. (2020). Effect of copayment on dental visits: A regression discontinuity analysis. Journal of Dental Research, 99(12), 1356–1362. 10.1177/0022034520946022 32735476

[cre2680-bib-0007] Guy, R. , Hocking, J. , Wand, H. , Stott, S. , Ali, H. , & Kaldor, J. (2012). How effective are short message service reminders at increasing clinic attendance? A meta‐analysis and systematic review. Health Services Research, 47(2), 614–632. 10.1111/j.1475-6773.2011.01342.x 22091980 PMC3419880

[cre2680-bib-0008] Hajek, A. , Kretzler, B. , & König, H.‐H. (2021). Factors associated with dental service use based on the andersen model: A systematic review. International Journal of Environmental Research and Public Health, 18(5), 2491. 10.3390/ijerph18052491 33802430 PMC7967618

[cre2680-bib-0009] Kannisto, K. A. , Koivunen, M. H. , & Välimäki, M. A. (2014). Use of mobile phone text message reminders in health care services: A narrative literature review. Journal of Medical Internet Research, 16(10), e222. 10.2196/jmir.3442 25326646 PMC4211035

[cre2680-bib-0010] Kaprio, H. , Suominen, A. L. , & Lahti, S. (2012). Association between subjective oral health and regularity of service use: Service use and subjective oral health. European Journal of Oral Sciences, 120(3), 212–217. 10.1111/j.1600-0722.2012.00955.x 22607337

[cre2680-bib-0011] Manning, W. G. , Bailit, H. L. , Benjamin, B. , & Newhouse, J. P. (1985). The demand for dental care: Evidence from a randomized trial in health insurance. The Journal of the American Dental Association, 110(6), 895–902. 10.14219/jada.archive.1985.0031 3894470

[cre2680-bib-0012] Mueller, C. D. , & Monheit, A. C. (1988). Insurance coverage and the demand for dental care. Journal of Health Economics, 7(1), 59–72. 10.1016/0167-6296(88)90005-7 10288442

[cre2680-bib-0013] Nguyen, L. , Häkkinen, U. , & Rosenqvist, G. (2005). Determinants of dental service utilization among adults—The case of Finland. Health Care Management Science, 8(4), 335–345. 10.1007/s10729-005-4143-7 16379416

[cre2680-bib-0014] Perry, J. G. (2011). A preliminary investigation into the effect of the use of the short message service (SMS) on patient attendance at an NHS dental access centre in Scotland. Primary Dental Care, os18(4), 145–149. 10.1308/135576111797512810 21968040

[cre2680-bib-0015] Prasad, S. , & Anand, R. (2012). Use of mobile telephone short message service as a reminder: The effect on patient attendance. International Dental Journal, 62(1), 21–26. 10.1111/j.1875-595X.2011.00081.x 22251033 PMC9374977

[cre2680-bib-0016] Raittio, E. (2016). Use of oral health services and perceived oral health after the oral health care reform introduced during 2001–2002: The more comprehensive public coverage of oral health care, the lower socioeconomic inequalities? (p. 50). University of Eastern Finland.

[cre2680-bib-0017] Raittio, E. , Kiiskinen, U. , Helminen, S. , Aromaa, A. , & Suominen, A. L. (2014). Dental attendance among adult Finns after a major oral health care reform. Community Dentistry and Oral Epidemiology, 42(6), 591–602. 10.1111/cdoe.12117 24954558

[cre2680-bib-0018] Riordain, R. N. , Glick, M. , Mashhadani, S. S. A. A. , Aravamudhan, K. , Barrow, J. , Cole, D. , Crall, J. J. , Gallagher, J. E. , Gibson, J. , Hegde, S. , Kaberry, R. , Kalenderian, E. , Karki, A. , Celeste, R. K. , Listl, S. , Myers, S. N. , Niederman, R. , Severin, T. , Smith, M. W. , … Williams, D. M. (2021). Developing a standard set of patient‐centred outcomes for adult oral health—An international, cross‐disciplinary consensus. International Dental Journal, 71(1), 40–52. 10.1111/idj.12604 33616051 PMC9275363

[cre2680-bib-0019] Schwebel, F. J. , & Larimer, M. E. (2018). Using text message reminders in health care services: A narrative literature review. Internet Interventions, 13, 82–104. 10.1016/j.invent.2018.06.002 30206523 PMC6112101

[cre2680-bib-0020] Stormon, N. , Sexton, C. , Chen, C. , Hsu, E. , Chen, P. C. , & McGowan, K. (2022). SMS reminders to improve outpatient attendance for public dental services: A retrospective study. Health & Social Care in the Community, 30(5), 2255. 10.1111/hsc.13663 34850473

[cre2680-bib-0021] Storrs, M. J. , Ramov, H. M. , & Lalloo, R. (2016). An investigation into patient non‐attendance and use of a short‐message reminder system at a University Dental Clinic. Journal of Dental Education, 80(1), 30–39. 26729682

[cre2680-bib-0022] Suominen, A. L. , Helminen, S. , Lahti, S. , Vehkalahti, M. M. , Knuuttila, M. , Varsio, S. , & Nordblad, A. (2017). Use of oral health care services in Finnish adults—Results from the cross‐sectional health 2000 and 2011 surveys. BMC Oral health, 17(1), 78. 10.1186/s12903-017-0364-7 28438160 PMC5402661

[cre2680-bib-0023] Tennant, P. W. G. , Murray, E. J. , Arnold, K. F. , Berrie, L. , Fox, M. P. , Gadd, S. C. , Harrison, W. J. , Keeble, C. , Ranker, L. R. , Textor, J. , Tomova, G. D. , Gilthorpe, M. S. , & Ellison, G. T. H. (2021). Use of directed acyclic graphs (DAGs) to identify confounders in applied health research: Review and recommendations. International Journal of Epidemiology, 50(2), 620–632. 10.1093/ije/dyaa213 33330936 PMC8128477

[cre2680-bib-0024] Torppa‐Saarinen, E. , Tolvanen, M. , Suominen, A. L. , & Lahti, S. (2018). Changes in perceived oral health in a longitudinal population‐based study. Community Dentistry and Oral Epidemiology, 46(6), 569–575. 10.1111/cdoe.12393 29956841

[cre2680-bib-0025] Torppa‐Saarinen, E. , Suominen, A. L. , Lahti, S. , & Tolvanen, M. (2019). Longitudinal pathways between perceived oral health and regular service use of adult Finns. Community Dentistry and Oral Epidemiology, 47(5), 374–380. 10.1111/cdoe.12478 31179572

[cre2680-bib-0026] Torppa‐Saarinen, E. , Tolvanen, M. , Lahti, S. , & Suominen, A. L. (2021). Changes and determinants of unmet oral health treatment need. Community Dentistry and Oral Epidemiology, 49(2), 158–165. 10.1111/cdoe.12587 33104256

[cre2680-bib-0027] Venables, W. N. , & Ripley, B. D. (2002). Modern applied statistics with S (4 ed., p. 498). Springer.

[cre2680-bib-0028] Wickham, H. (2016). ggplot2: Elegant graphics for data analysis (p. 260). Springer‐Verlag.

[cre2680-bib-0029] Yoshida, K. , & Bartel, A. (2021). *Tableone: Create ‘Table 1’ to describe baseline characteristics with or without propensity score weights*. Accessed November 21, 2021. Retrieved from https://CRAN.R-project.org/package=tableone

