# Supplementary figures and images for: Association of service use with subjective oral health indicators in a freedom of choice pilot
Source: Clin Exp Dent Res. 2022 Oct 20;9(1):134–41. doi: 10.1002/cre2.680 (PMC9932228; doi:10.1002/cre2.680)

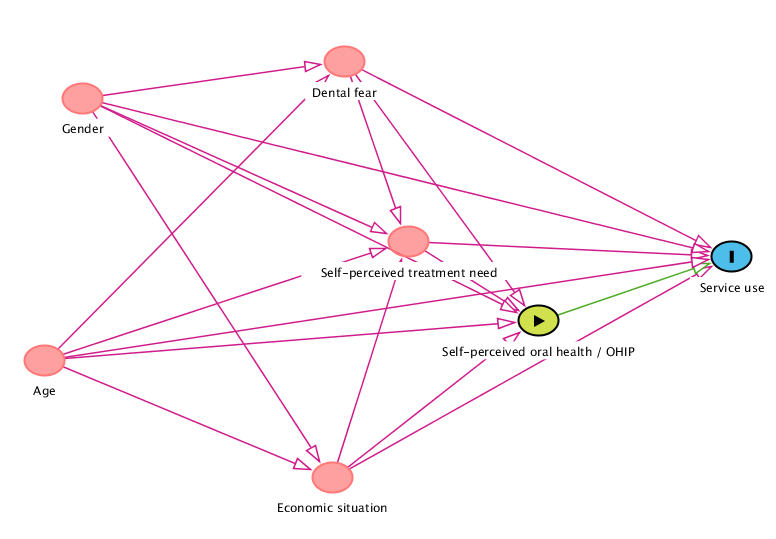

Supplement: Supplementary file 1 — Supporting information. [file CRE2-9-134-s001.png]
